# Integrative proteomics to understand the transmission mechanism of *Barley yellow dwarf virus-GPV* by its insect vector *Rhopalosiphum padi*

**DOI:** 10.1038/srep10971

**Published:** 2015-07-10

**Authors:** Hui Wang, Keke Wu, Yan Liu, Yunfeng Wu, Xifeng Wang

**Affiliations:** 1 State Key Laboratory for Biology of Plant Diseases and Insect Pests, Institute of Plant Protection, Chinese Academy of Agricultural Sciences, Beijing, China; 2 College of Plant Protection, Northwest A & F University, Yangling 712100, China

## Abstract

*Barley yellow dwarf virus-GPV* (BYDV-GPV) is transmitted by *Rhopalosiphum padi* and *Schizaphis graminum* in a persistent nonpropagative manner. To improve our understanding of its transmission mechanism by aphid vectors, we used two approaches, isobaric tags for relative and absolute quantitation (iTRAQ) and yeast two-hybrid (YTH) system, to identify proteins in *R. padi* that may interact with or direct the spread of BYDV-GPV along the circulative transmission pathway. Thirty-three differential aphid proteins in viruliferous and nonviruliferous insects were identified using iTRAQ coupled to 2DLC-MS/MS. With the yeast two-hybrid system, 25 prey proteins were identified as interacting with the readthrough protein (RTP) and eight with the coat protein (CP), which are encoded by BYDV-GPV. Among the aphid proteins identified, most were involved in primary energy metabolism, synaptic vesicle cycle, the proteasome pathway and the cell cytoskeleton organization pathway. In a systematic comparison of the two methods, we found that the information generated by the two methods was complementary. Taken together, our findings provide useful information on the interactions between BYDV-GPV and its vector *R. padi* to further our understanding of the mechanisms regulating circulative transmission in aphid vectors.

The Barley yellow dwarf viruses (BYDVs, genus *Luteovirus* or *Polerovirus*, family *Luteoviridae*) are economically important pathogens of wheat, barley, maize, oat and pasture grasses in the world^1^. They are phloem-limited viruses transmitted by different cereal aphids in a persistent nonpropagative manner^2^. At least 25 aphid species have been reported as vectors of BYDVs, but each virus is transmitted by only one or few aphid species^3^,^4^. In China, four BYDV species have been reported, namely, BYDV-GAV, -GPV, -PAV, and -RMV, respectively^5^,^6^,^7^. Of these four, BYDV-GPV, which is transmitted efficiently by *Rhopalosiphum padi* and *Schizaphis graminum*, is treated as an unassigned member of the family *Luteoviridae* in the most recent ICTV’s Virus Taxonomy Report^8^. The complete nucleotide sequence of BYDV-GPV was determined in 2009; its genome comprises 5673 nucleotides with six predicted open reading frames (ORFs) and three untranslated regions (UTRs), similar to the genome of poleroviruses^9^. Comparisons between different open reading frames (ORFs) of the genomes of BYDV-GPV, other poleroviruses and luteoviruses demonstrated that the virus encodes two structural proteins, the major coat protein (CP) and the readthrough protein (RTP), which are responsible for composition of the viral capsid and playing an important role in transmission by the aphids^9^,^10^.

Early work on BYDV transmission by aphids focused on the description of primary aphid species based on transmission efficiency and quantitative parameters, such as the time required for virus acquisition by an aphid on an infected plant, the length of time the infectious virus is retained, and the time required for efficient transmission into a new healthy plant^3^,^4^. This early work was followed by extensive electron microscopy studies on the transport pathway of the virus within the aphid vectors^2^,^11^. The virus crosses the gut epithelium at the posterior midgut and/or hindgut level via transcytosis and exits these cells by exocytosis to enter the hemocoel^12^,^13^. Once released from the posterior midgut and/or hindgut, BYDVs are believed to diffuse passively into the hemolymph until they encounter putative receptors located specifically at the basal lamina of the salivary gland cells. They then invade the salivary gland cells, also involving endocytosis and exocytosis, from where they will be introduced into the plant host during insect feeding^14^,^15^. So, the viruses must encounter and overcome different barriers in the posterior midgut and/or hindgut and salivary gland cells for successful persistent transmission; thus, specific interactions between components of the virus and its vector are necessary^16^.

Recent investigations have resulted in a better understanding of the virus and aphid proteins involved in overcoming transmission barriers in the aphid vectors. In the family *Luteoviridae*, both the CP and RTP have been implicated in aphid transmission^17^,^18^. Several proteins of *Myzus persicae* were found to bind to the virion of *Beet western yellows virus*, including the receptor for activated C kinase (Rack-1), actin, and glyceradehyde-3-phosphate dehydrogenase (GAPDH3), which may be involved in the epithelial transcytosis of virus particles in the aphid vectors^19^. Two proteins, SaM50 and SaM35 from *Sitobion avenae* and *S. graminum* showed high affinity for BYDV-MAV or -GAV and contributed to viral transmission specificity^20^,^21^. Four aphid proteins, including luciferase and cyclophilin, which have been implicated in macromolecular transport, were found to be specifically associated with the ability of *S. graminum* to transmit *Cereal yellow dwarf virus* (CYDV)-*RPV*^22^,^23^. Cilia *et al*. (2011)^24^ used 2-D fluorescence difference gel eletrophoresis (DIGE) of proteins from an F_2_ generation derived from hybrids between clones of *S. graminum* that differed in transmission efficiency for CYDV-RPV to identify proteins correlated with a transmission phenotype that was stably inherited and expressed in the absence of the virus. They found that the specificity of virus transmission by aphids was due to quantitative and heritable proteomic variation and possibly derived from allele-specific variation in the genetic loci encoding for these proteins^24^.

Although new advances have been made in our understanding of the transmission mechanism of viruses in the family *Luteoviridae* by their respective aphid vectors, few studies have focused specifically on the interaction between BYDV-GPV and its insect vector *R. padi*. In recent years, some new techniques have been used to study the interaction between the host, pathogen and vector. Isobaric tags for relative and absolute quantitation (iTRAQ) is one platform for quantitative proteomics to compare changes in the abundance of specific proteins between different samples^25^,^26^. The yeast two-hybrid (YTH) system is also proving to be a powerful tool for proteomic-based investigations and has become a pivotal tool to study uncharacterized protein-protein interactions^27^,^28^. In this study, we took advantage of the two approaches to identify proteins in *R. padi* that may interact with and/or mediate the spread of BYDV-GPV in the aphid vectors. A comparison of the data obtained by two methods could also provide useful information regarding intrinsic merits and constraints of these methods.

## Results

### Identification of differential proteins in viruliferous and nonviruliferous *Rhopalosiphum padi* using iTRAQ

**Figure 1:**
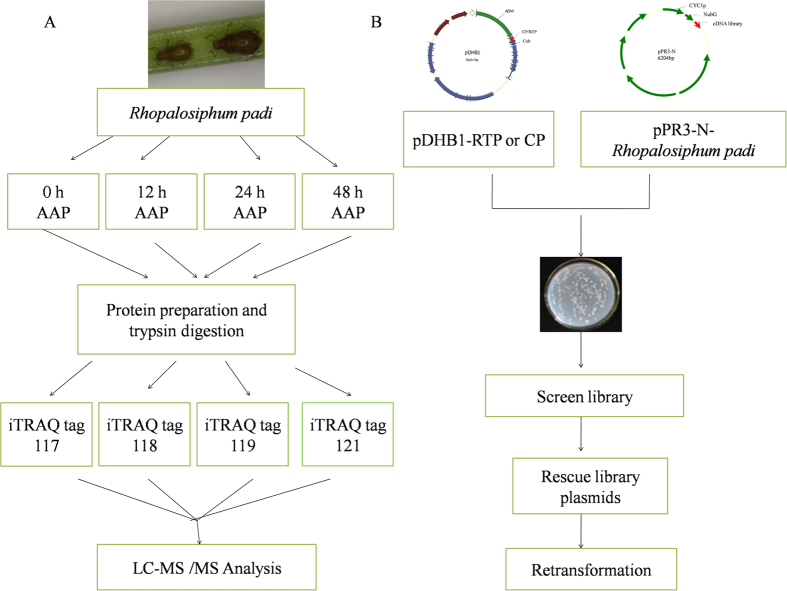
Schematic of iTRAQ design (**A**) and yeast two-hybrid screen design (**B**). iTRAQ experiment was carried out using the vector aphids *Rhopalosiphum padi*, which were allowed a 0, 12, 24 or 48 h acquisition access period (AAP) on BYDV-GPV-infected oat plants. Proteins were digested by trypsin, and four iTRAQ labels were used: tag 117 for the 0 h AAP, tag 118 for the 12 h AAP, tag 119 for the 24 h AAP and tag 121 for the 48 h AAP. After labeling, peptides from all four samples were combined and fractionated by chromatography. Each fraction was then analyzed by LC-MS/MS. For yeast two-hybrid screen, the ORFs of BYDV-RTD/BYDV-CP were constructed individually in the yeast expression vector pDHB1, and the cDNA library of *R. padi* was constructed in prey plasmid pPR3-N. The cDNA library of *R. padi* was then screened and positive plasmids were rescued. The prey plasmid and bait plasmid were then used to cotransform yeast strain NMY51 to eliminate false positive hits.

Proteins from samples of aphids with 0 h, 12 h, 24 h and 48 h acquisition access period (AAP) on oat plants infected with BYDV-GPV were identified and quantified using 4 plex iTRAQ labeling combined with LC-MS/MS analysis (Fig. 1). In total, we identified 628 proteins, with false discovery rates (FDR) less than 0.01 for the sample sets. These identified proteins were then filtered using population statistics to obtain a list of proteins that differed significantly between the viruliferous and healthy samples. Proteins with a 50% increase or decrease in abundance (iTRAQ ratios greater than 1.5 or less than 0.667) and a significant *p*-value (*p* < 0.05) were considered to be significantly differential. A strict cutoff value of a 1.5-fold change resulted in a final set of 33 differential proteins. A complete list of proteins identified in this study, and the details on the proteomic data, including sequence coverage, number of unique peptides, ratios between different samples, and functional pathways, are listed in Table 1.

**Figure 2:**
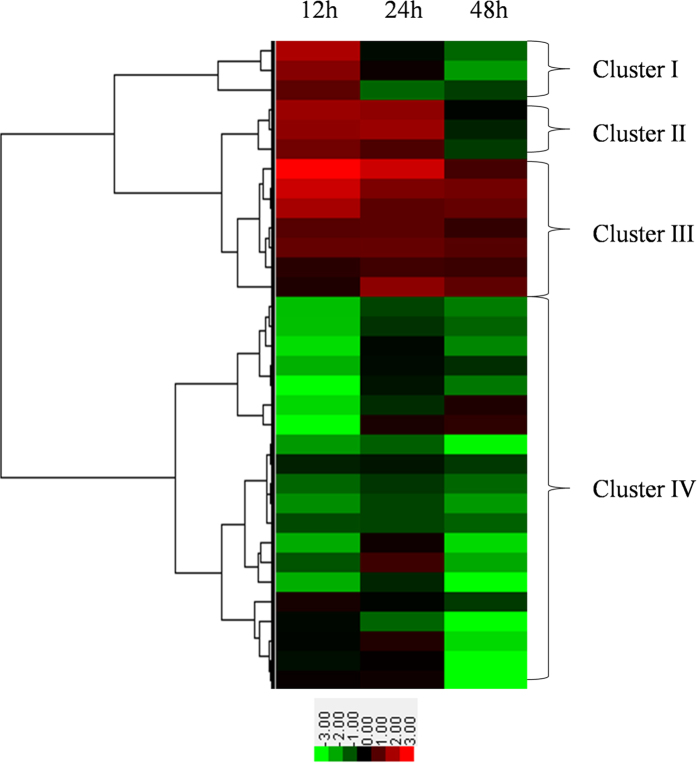
Cluster analysis of differential proteins by hierarchical clustering algorithm. The three columns from left to right are labeled 12, 24 and 48 h AAP at the top of the heat map. The right braces indicate the four groups classified by cluster analysis. The lower panel is the color key for fold-change. Red represents up-regulation, green represents down-regulation; the brighter the image, the greater the fold-change.

Using the entire set of quantitative data, we further characterized changes in abundance of these differential proteins. We performed cluster analysis by combining the protein abundance data and the sampling time points and using a hierarchical clustering algorithm according to the program instructions. With the proteome of a nonviruliferous aphid as a reference, the 33 differential proteins were clustered into 4 clusters (Fig. 2). Only 3 proteins grouped in cluster I and 3 in cluster II. The abundance of the proteins in cluster I increased after a 12 h acquisition access period (AAP) but subsequently decreased. Proteins in cluster II increased in abundance beginning at 12 h AAP, continued to increase in abundance through 24 h AAP, then decreased. These proteins were likely to be active only during a specific process. Most of the proteins, however, belonged to cluster III and cluster IV. Seven proteins in cluster III were always upregulated in viruliferous aphids compared with nonviruliferous aphids after 12 h, 24 h and 48 h AAP. The largest group was cluster IV, which contained 20 proteins that in general decreased in abundance.

**Figure 3:**
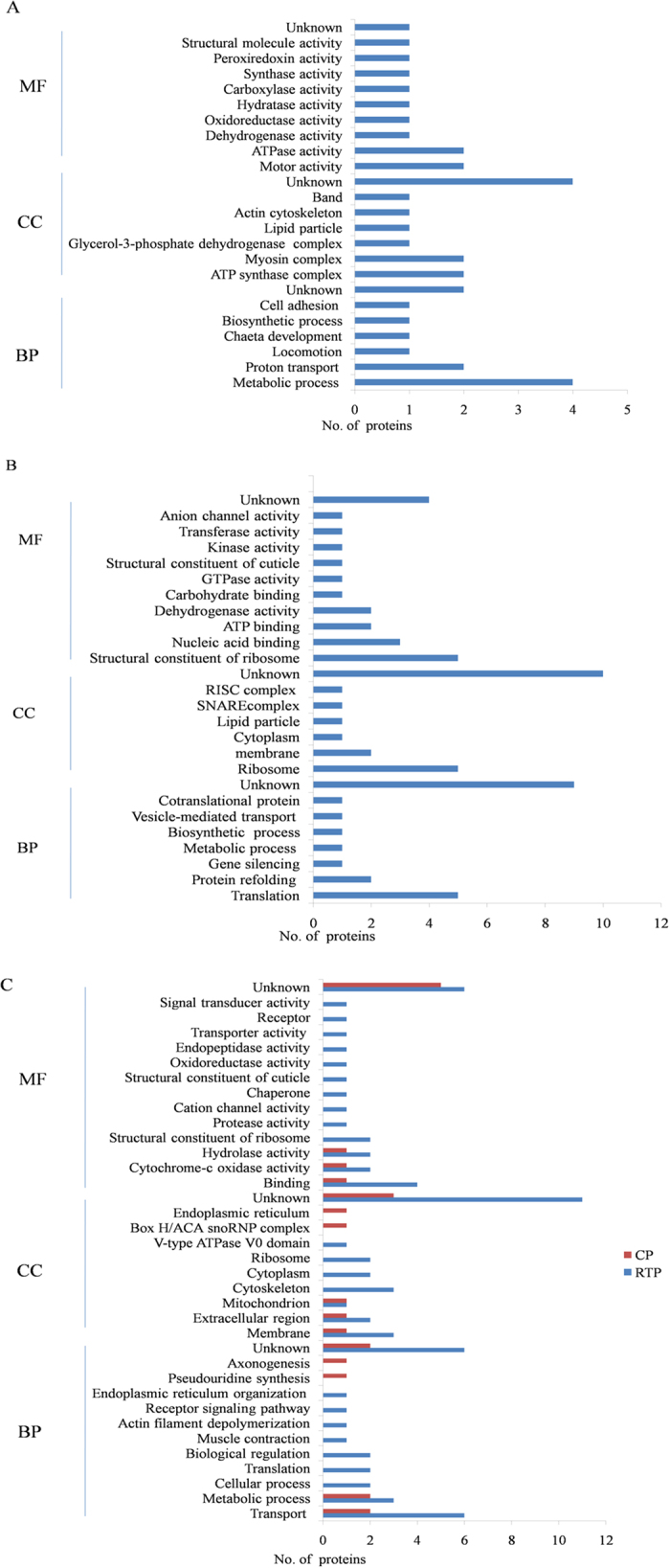
Functional annotation of (**A**) up-regulated proteins, (**B**) down-regulated proteins identified with iTRAQ and (**C**) proteins identified from yeast two-hybrid screen. Three main categories were identified: biological process (BP), cellular component (CC) and molecular function (MF). The *x*-axis represents the number of proteins.

**Figure 4:**
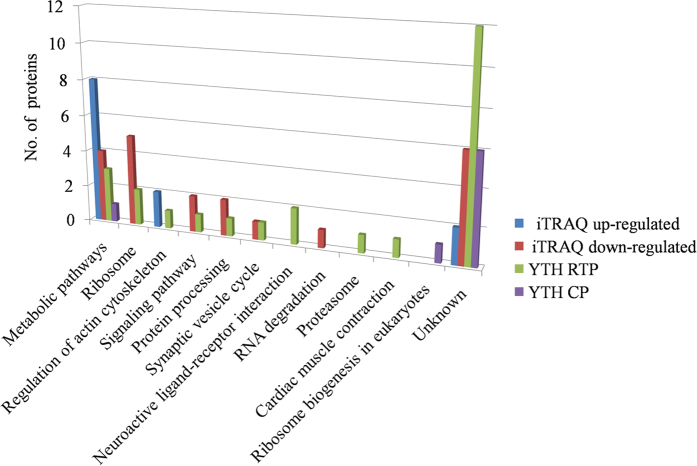
Pathway analysis of proteins identified by iTRAQ and YTH. The *y*-axis represents the number of proteins.

All the differential proteins were also functionally annotated according to biological process, cellular component and molecular function using Uniprot (http://www.uniprot.org) and DAVID (http://david.abcc. ncifcrf.gov/). The prevalent biological processes of the up-regulated proteins were metabolic processes, proton transport and locomotion, while down-regulated proteins, most were in translation, gene silencing and protein refolding. The most up-regulated proteins were cellular components of ATP synthase complex and myosin complex, while down-regulated proteins were assigned to ribosome, membrane and SNARE complex. The molecular functions of up-regulated proteins were motor activity and enzymatic activity, while major functional categories of down-regulated proteins were structural molecule activity, binding activity and dehydrogenase activity. (Fig. 3A,B). The pathways analysis of these differential proteins using the Kyoto Encyclopedia of Genes and Genomes (KEGG) database (http://www.kegg.jp/kegg/pathway.html) showed that up-regulated proteins basically involved in regulation of actin cytoskeleton and energy and primary metabolism, while down-regulated proteins participated mainly in ribosome, signaling pathway, RNA degradation and synaptic vesicle cycle (Fig. 4).

### Verification of mRNA expression level for three representative proteins (myosin, paramyosin and vinculin) by RT-qPCR

**Figure 5:**
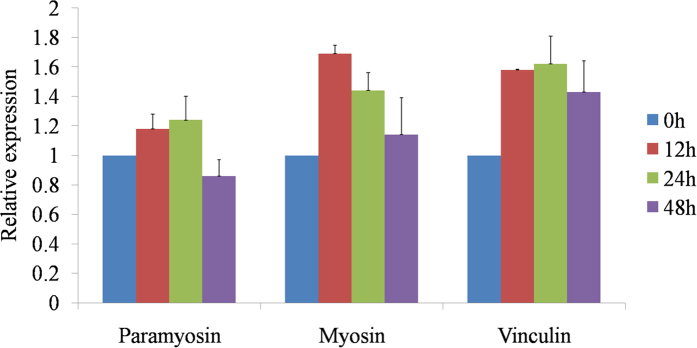
RT-qPCR validation at mRNA transcription level of iTRAQ abundance results for three proteins (myosin, paramyosin and vinculin).

To further confirm the results of iTRAQ and compare the correlation between mRNA transcription level and protein abundances, three representative proteins (myosin, paramyosin and vinculin) were selected based on the functional annotation and verified for mRNA transcription level by RT-qPCR. Myosin and vinculin were always up-regulated after 12, 24 and 48 h AAP. Paramyosin was up-regulated after 12 and 24 h AAP and down-regulated after 48 h AAP. These results for mRNA transcription level for the selected genes showed the same trends as in the results obtained with the iTRAQ analysis (Fig. 5).

### Interactions of the proteomes between *Barley yellow dwarf virus*-*GPV* and its insect vector, *Rhopalosiphum padi*, by yeast two-hybrid assay

In the present study, we identified protein interactions between BYDV-GPV using RTP or CP as the bait and the cDNA library of *R. padi* as the prey in a yeast two-hybrid system (Fig. 1B). To confirm that the bait fusion protein was functionally well expressed in the assay without auto-activation activity, each set of plasmids was used to cotransform NMY51 cells, which were then plated on selective SD medium (synthetic double-dropout medium [DDO: SD/-Leu/-Trp]/synthetic triplex-dropout medium [TDO: SD/-His /-Leu/-Trp]/synthetic quadruple-dropout medium [QDO: SD/−Ade/−His/−Leu/−Trp]). The result of the co-expression (pDHB1-RTP or pDHB1-CP bait vector with the control prey plasmid Ost1-NubI) was the same as the positive control. This set of plasmids enables the expression of reporter genes and thus growth on QDO selective medium. Coexpression of the bait protein with the NubG-nonsense peptide fusion vector (pPR3-N fusion vector) did not result in split ubiquitin formation; thus no yeast transformants grew on QDO plate (Supplementary Figure S1).

In the preliminary library screen of the pDHB1-RTP bait/pPR3-N-*R.padi* cDNA prey, we obtained 350 yeast colonies from TDO medium and 280 from pDHB1-CP bait/pPR3-N-*R.padi* cDNA prey. When the potentially positive clones that exhibited bait protein-protein interactions were taken from the TDO medium and streaked onto QDO medium, 87 clones acting as prey proteins grew on QDO medium from the pDHB1-RTP bait, and 46 clones acting as prey proteins grew on QDO medium from the pDHB1-CP bait. All sequences were used to search for reference sequences using BLASTX against the nonredundant (nr) NCBI protein database (http://blast.ncbi.nlm.nih.gov/Blast.cgi). We identified 25 prey proteins that interacted with RTP of BYDV-GPV and eight that interacted with the CP of the virus, and five prey proteins that were common to the two bait groups. A complete list of proteins identified in this assay, and the details of BLAST data including e-value, identity and pathway are listed in the Table 2.

For the Gene Ontology (GO) annotation using the same database, 25 proteins screened using the BYDV-GPV RTP bait were assigned to 9 categories of biological process, mainly for transport, metabolic process, muscle contraction and actin filament depolymerization; 7 categories of cellular components, mainly in membranes, extracellular region and cytoskeleton; and 13 categories of molecular function, mainly with binding activity, cytochrome-c oxidase activity and transporter activity (Fig. 3C). In the pathways analysis of these proteins using the KEGG database, the proteins were basically involved in metabolic pathways, ribosome, regulation of actin cytoskeleton and synaptic vesicle cycle (Fig. 4). Eight proteins screened using the BYDV-GPV CP bait were assigned to 4 categories of biological process, mainly for transport and metabolic process; 5 categories of cellular components, mainly in membranes and extracellular region; and 3 categories of molecular function, mainly with binding activity and cytochrome-c oxidase activity (Fig. 3C). They were basically involved in metabolic pathways and ribosome biogenesis in eukaryotes (Fig. 4).

### Verification of the yeast two-hybrid results using a retransformation assay and chemiluminescent CO-IP assay

**Figure 6:**
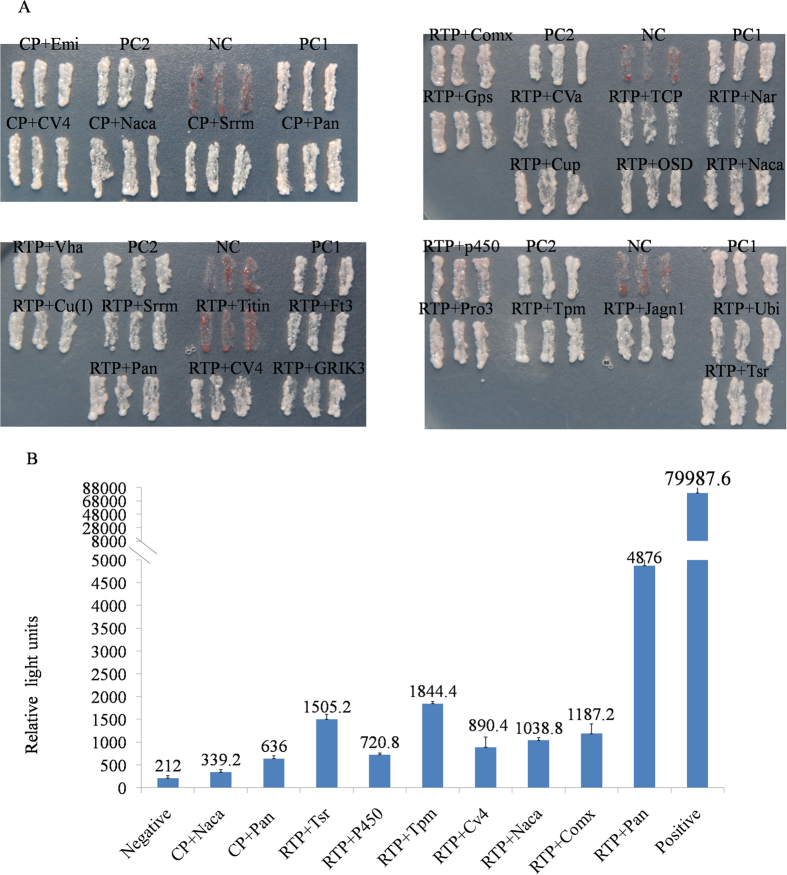
(**A**) Confirmation of protein-protein interactions by retransformation assay. Bait plasmids (pDHB1-GPV-RTP or pDHB1-GPV-CP bait plasmids) were cotransformed with the prey fusion proteins in yeast strain NMY51. Potential interactions were assessed by plating the transformants on SD/-Ade/-His/-Leu/-Trp (QDO) medium and incubating them for 3-4 days at 30 °C. (**B**) Confirmation of protein-protein interactions using a chemiluminescent Co-IP assay. We examined the interactions of the BYDV-GPV RTP bait and the BYDV-GPV CP bait with positive prey proteins in HEK293T mammalian cells. After substrate addition, the relative strength and interaction of the resulting signal for pProLabel activity were measured with a GloMax 96 Microplate Luminometer and expressed as relative light units. Protein abbreviations. Emi: EMI domain-containing protein, CV4: cytochrome c oxidase polypeptide IV-like, Naca: nascent polypeptide-associated complex subunit alpha, Srrm: serine/arginine repetitive matrix, Pan: pancreatic lipase-related protein, Comx: complexin, Gps: guanine nucleotide-binding protein G(s) subunit alpha, CVa: cytochrome c oxidase subunit Va-like, TCP: T-complex protein 1 subunit theta-like, Nar: neuronal acetylcholine receptor subunit alpha-7-like, Cup: cuticular protein 5 precursor, OSD: chemosensory protein-like precursor, Vha: V-type proton ATPase subunit d, Cu(I): Cu(I)-responsive transcriptional regulator, Titin: titin-like, Ft3: fibronectin type 3 domain, GRIK3: glutamate receptor ionotropic kainate 3-like isoform X3, p450: cytochrome P450 4g15, Pro3: proteasome beta 3 subunit-like, Tpm: tropomyosin, Jagn1: jagunal homolog, Ubi: ubiquitin carboxyl-terminal hydrolase, Tsr : twinstar.

To eliminate false positive hits and retest the specificity of the interactions, we did retransformation confirmation assays. We selected 22 candidate prey plasmids screened with the pDHB1-RTP bait and 5 screened with the pDHB1-CP bait for the interaction analysis. We thus identified 19 proteins that interacted strongly with pDHB1-RTP, 2 prey proteins (T-complex protein 1 subunit theta-like and cytochrome c oxidase subunit Va-like) interacted weakly with pDHB1-RTP, and 1 prey protein (titin) did not interact with pDHB1-RTP. We identified 5 proteins that interacted strongly with pDHB1-CP (Fig. 6A).

The interaction between BYDV-GPV and positive prey proteins was further assessed in a chemiluminescent Co-IP assay using mammalian cells. In this experiment, combinations of pAcGFP-Lam/pProlabe-T were used as the negative control and pAcGFP-p53/pProLabel-T were used as the positive control. The relative strengths of the interactions between the BYDV-GPV RTP and 7 prey proteins (pancreatic lipase-related protein, tropomyosin, twinstar, complexin, nascent polypeptide-associated complex subunit alpha, cytochrome c oxidase polypeptide IV-like and cytochrome P450 4g15) were 23, 8.7, 7.1, 5.6, 4.9, 4.2, and 3.4 times greater, respectively, than the negative control, and the relative strengths of the interactions between the BYDV-GPV CP and 2 prey proteins (pancreatic lipase-related protein, nascent polypeptide-associated complex subunit alpha) were 3 and 1.6 times, respectively, greater than the negative control. These results confirmed those of the yeast two-hybrid assay and indicated the interaction strength between bait protein and various candidate prey proteins (Fig. 6B).

### Comparative proteomics of the data obtained by iTRAQ and yeast two-hybrid system

In this study, we used iTRAQ and YTH to identify various proteins in *R. padi* that may interact with BYDV-GPV. These methods were compared for their characterization of the proteomes involved in the BYDV-GPV-*R. padi* interaction for future use in more targeted studies on the mechanisms regulating circulative virus transmission in aphids. In total, we identified 28 potential protein-protein interactions using YTH and 33 differential proteins using iTRAQ. Of all proteins, except two homologous proteins (cuticular protein 5 precursor and cuticular protein 62 precursor) were identified by YTH and iTRAQ, respectively, others are different proteins. The pathways of these proteins are thus the main focus because the proteins may work together for virus transmission. All proteins were distributed among 11 categories of pathways in the KEGG database, and six of these pathways were shared by the 33 proteins that were identified by both methods. Of these 33 proteins, 15 (12 from iTRAQ and three from YTH) were involving in metabolic pathways, seven (five from iTRAQ and two from YTH) in ribosomes, three (two from iTRAQ and one from YTH) in regulation of the actin cytoskeleton, three (two from iTRAQ and one from YTH) in signal pathways, three (two from iTRAQ and one from YTH) in protein processing, and two (one from iTRAQ and one from YTH) in the synaptic vesicle cycle (Fig. 4; Table 1 and 2).

**Figure 7:**
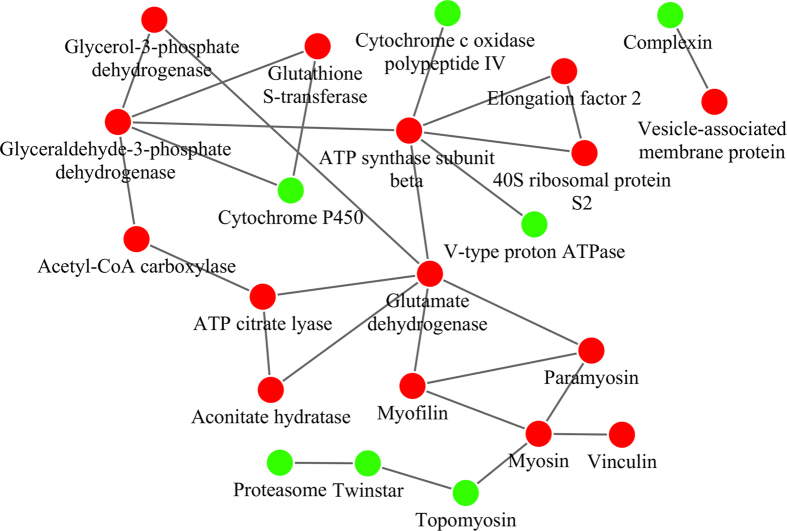
Protein-protein interaction network analysis. This interaction network for all proteins identified by two methods was created using String. Orphan proteins, i.e., unconnected proteins, were removed, and the network information was exported for visualization in Cytoscape v.3.1.1. Red nodes represent proteins identified by iTRAQ, green nodes represent proteins identified by yeast two-hybrid screen.

Several proteins related to metabolic pathways, regulation of the actin cytoskeleton and the synaptic vesicle cycle were identified from both iTRAQ and YTH. Thus, we created a network based on known protein-protein interactions using the STRING database (http://string-db.org) and imported the proteomic data into the Cytoscape application v.3.1.1 (http://www.cytoscape.org) (Fig. 7). Among the 61 proteins identified by the two methods, 22 proteins were included in the network (15 identified by iTRAQ, 7 by YTH). Green nodes represent proteins identified by YTH, and red nodes represent proteins identified by iTRAQ. This network suggested that the results of the two methods are complementary. The protein interaction network showed that vesicle-associated membrane protein, which is involved in the synaptic vesicle cycle^29^, is directly linked to complexin. Complexin positively regulates synaptic vesicle exocytosis by acting on transmembrane regions of syntaxin and vesicle-associated membrane protein^30^. Overall, the network highlighted particular importance for the host cytoskeleton, including myosin, topomyosin and others, which are implicated in the movement of plant and animal viruses^31^.

## Discussion

The circulative transmission pathway through an aphid vector involves complex interactions between viral proteins and vector-associated compounds^2^,^32^. Quantitative proteomics has been used to study vector-virus interactions for decades and is still widely used. Vector proteins that interact with a virus have been identified by two-dimensional gel electrophoresis and LC/MS^24^, and a specific interaction between ribosomal protein S2 of *M. persicae* and *Tobacco etch virus* HC-Pro was confirmed by 2D electrophoresis separation and far western blotting^33^. In another study, coupling of differential proteins with the transmission phenotype (*S. graminum*) was investigated using a 2D electrophoresis quantitative proteomic approach^24^. However, accurate quantification using 2D electrophoresis is sometimes compromised by comigration or partial comigration of proteins^34^,^35^.

iTRAQ is a fairly recent quantitative proteomics technique that is gradually gaining popularity. The iTRAQ technology label isobaric reagents at the N termini and lysine side chains of peptides in a digest mixture and yield reporter ions following MS that can be used to identify and quantify the abundance differences of the proteins in tested samples^36^,^37^. This approach simplifies analysis and will potentially increase analytical accuracy and precision^38^,^39^. Using this technology, we identified 33 differential proteins between healthy and viruliferous insects. Another frequently-used method in proteomic study, YTH assay has been used extensively to detect protein-protein interactions. When a C-terminal fragment of ubiqultin (Cub) is expressed as a fusion to a reporter protein, the fusion is cleaved and reporter protein expresses only if a N-terminal fragment of ubiquin (Nub) is also expressed in the same cell^40^. Based on this principle, we linked BYDV-GPV CP/RTP bait to Cub and cDNA library of *R. padi* to mutationally altered Nub and screened vector proteins that interacted with virus bait directly. Through YTH screening of a rice cDNA library, Kong *et al*. (2014)^41^ found that the disease-specific protein of *Rice stripe virus* (RSV) interacted with rice PsbP (oxygen-evolving complex protein), which plays an important role on symptom development. Vitellogenin (Vg) of *Laodelphax striatellus* interacts strongly with the nucleocapsid protein of RSV in a YTH assay and was found to play a critical role in carrying the virion into the ovary of the vectors^42^. We identified 25 prey proteins that interacted with BYDV-GPV RTP and eight prey proteins that interacted with BYDV-GPV CP using YTH assay. Nevertheless, other interactions might not be detected in YTH assay because of the lack of chaperones, assembly factors, post-translational modifications, or other effects^43^. In addition, the YTH assay may produce false positives if (1) the two proteins are not expressed in the same tissue or at the same time or if (2) the prey protein has a self-activating function^43^,^44^.

Using the two methods, we identified 61 proteins that might be involved in the transmission of BYDV-GPV by *R. padi*. In a previous study using 2D DIGE, Cilia *et al*. (2011)^24^ the 50 differential proteins identified in the vector aphid *S. graminum* may be involved in energy metabolism, membrane trafficking, lipid signaling, and the cytoskeleton and bacterial endosymbiosis. Proteins homologous to those identified by Cilia *et al*.^24^ were also identified in our study, including glyceraldehyde-3-phosphate dehydrogenase, ATP synthase subunit beta, cuticular protein, peroxiredoxin (using iTRAQ) and the cuticular protein and proteasome subunit beta (using the YTH assay).

The proteasome, a large multi-subunit proteinase complex found in the cytoplasm and nucleus of all eukaryotic cells, fulfills vital cellular functions, including elimination of misfolded proteins, generating antigenic peptides, degradation of nuclear and membrane-bound proteins, and regulation of important cellular processes^45^,^46^. The tat protein of *Human immunodeficiency virus*-1 (HIV-1) can interact with the proteasome *in vivo*, then block the proteolytic activity of the proteasome, thus contributing directly to virion escape from host immunity^47^,^48^. On the basis of our results and published reports, the proteasome of *R. padi* is strongly implicated as an antiviral immune response against the movement process of BYDV-GPV in the body of its aphid vectors.

In this study, we found that the cuticular protein not only interacted with BYDV-GPV RTP, but it was also differentially expressed between the viruliferous and the healthy aphids. The abundance of this protein changed significantly after uptake of the *Pea enation mosaic virus* by the pea aphid (*Acyrthosiphon pisum*) in a transcriptomic analysis of the aphid intestinal genes^49^. According to a proteomic analysis, several cuticle proteins were also differentially expressed in genotoyes of *S. graminum* that differed in their ability to transmit CYDV-RPV^24^, indicating that cuticle proteins may have functions in the hindgut barrier. Recently, our lab found cuticle proteins and pc3 of RSV colocalized in the hemolymph of the small brown planthopper (*L. striatellus*). When the gene encoding the cuticle protein is silenced by RNAi, the number of viral particles of the virus in the vector insects decreased simultaneously, then virus transmission efficiency by the vector also declined (Liu *et al*., unpublished manuscript, under review by Mol & Cell Proteomics). Cuticle proteins may aid in the spread of the virus into the hemolymph and protect the virus from degradation by a host immune response.

Vesicle-associated membrane protein (VAMP) and complexin, which are involved in vesicular trafficking and synaptic vesicle exocytosis^50^,^51^ were identified using iTRAQ and YTH assay, respectively. Although receptor-mediated trancytosis (endocytosis/exocytosis) has been proposed as a mechanism for regulating vector-specific transmission of BYDVs by their aphid vectors^52^, the vector proteins involved in the endocytosis/exocytosis are largely unknown.

The keystone of the vesicle cycle is Ca^2+^-regulated exocytosis, followed by different routes of endocytosis and recycling^29^. During the sequential steps of the synaptic vesicle cycle neurotransmitters are first actively transported into synaptic vesicles, which are clustered in front of the active zone. The synaptic vesicles then dock at the active zone, where the vesicles are primed and converted into a state of competence for Ca^2+^-regulated fusion-pore opening. After the fusion pore opens, the synaptic vesicles undergo endocytosis and are recycled^29^. It has been shown that soluble N-ethylmaleimide-sensitive factor attachment protein receptor (SNARE) proteins, which contain synaptobrevin (vesicle-associated membrane protein) on the vesicular membrane and syntaxin and SNAP-25 on the plasma membrane, play an essential role in Ca^2+^-regulated synaptic vesicle exocytosis^53^. The synaptic vesicle membrane and plasma membranes are forced into close proximity, and the fusion pore is opened by the SNARE complex assembly^29^,^30^. Human vesicle-associated membrane protein subtype A and subtype B, which participate in the regulation of membrane trafficking, lipid transport and metabolism, and the unfolded protein response, are known to associate with NS5B and NS5A of *Hepatitis C virus* (HCV)^54^. Vesicle-associated membrane protein is also essential for two fast synapse specific membrane-trafficking reactions: fast exocytosis for neurotransmitter release and fast endocytosis that mediates rapid reuse of synaptic vesicles by defective mice^50^.

Complexin, also known as synaphin, is a neuronal cytosolic protein that acts as a positive regulator of synaptic vesicle exocytosis. Complexin binds to a fully assembled SNARE complex strictly in the transmembrane regions of syntaxin and vesicle-associated membrane protein as shown using purified full-length and truncated SNARE proteins and a gel shift assay^30^. In mast cells, complexin II regulates exocytosis positively through its translocation to the plasma membrane and enhancement of the Ca^2+^ sensitivity of the fusion machinery^51^. In light of our results, a direct mechanism based on complexin and vesicle-associated membrane protein involvement in BYDV-GPV movement across the barrier in *R. padi* is reasonable.

The cytoskeleton, consisting mostly of microtubules, microfilaments and intermediate filaments, has important roles for many viruses in completing their life cycle^55^. Viruses can induce rearrangements of cytoskeletal filaments to utilize them as tracks or to move them aside when they are barriers^31^. In the present study, the cytoskeleton component tropomyosin, which interacted with BYDV-GPV RTP according to the YTH assay, and proteins such as myosin and paramyosin that have microfilament motor activity and ATPase activity according to the GO annotation, were up-regulated in viruliferous aphids. In the YTH and Co-IP assays, myosin light chain 2 in the vector *Sogatella furcifera* interacted with P7-1(a nonstructural protein which is the major component of the tubular structure in vector tissues) of SRBSDV^56^. The localization of myosin with Pns10 (a nonstructural protein of *Rice dwarf virus*) was also confirmed in an RDV-infected vector cell monolayer culture; myosin motors were required for the transport of Pns10 containing virus particles to neighboring cells^57^. Similarly, the vaccinia virus F11 protein interacts with the RhoGAP myosin-9A to inhibit RhoA signaling^58^. In the absence of myosin-9A experimentally, RhoA signaling is not inhibited, resulting in fewer actin tails and reduced virus release concomitant with the spread of fewer viral particles. Thus, these proteins in the vector may facilitate virions trafficking by binding to BYDV-GPV proteins.

In conclusion, our results suggest that the information generated by the iTRAQ and YTH methods is complementary. These identified proteins may provide useful information on the protein and functional interactions between BYDV-GPV and vector *R. padi* and will also facilitate our understanding of the molecular mechanism that enables BYDV-GPV to cross the barriers posed by the hindgut and the epithelial cells of the accessory salivary glands in insect vectors. The results obtained with these methods have provided us with exciting directions for future studies on protein function using RNA interference (RNAi) technology and on verifying the involvement of the proteins identified in virus transmission using confocal microscopy.

## Methods

### Virus maintenance and aphid rearing

Laboratory isolates of BYDV-GPV have been maintained on oat plants (*Avena sativa* K. Koch cv. Coast-Black) in our laboratory since the 1990s^59^. A laboratory population of nonviruliferous aphids of *R. padi* was reared on winter wheat (*Triticum aestivum* L., cv. Beijing 837), under controlled conditions at 18 °C in a growth chamber^60^.

### Isobaric tags for relative and absolute quantitation (iTRAQ) between viruliferous and nonviruliferous *Rhopalosiphum padi*

#### Sample collection

Approximately 240 wingless adult insects of *R. padi* were picked from wheat with a soft brush, and then released carefully onto oat plants infected with BYDV-GPV for acquisition of viruses. After each feeding duration (0 h, 12 h, 24 h, 48 h), 50 wingless adult aphids were collected.

### Sample preparation for proteomics and iTRAQ labeling

The sample was weighed and dissolved in 800 μL of lysis buffer (7 M urea, 2 M thiourea, 0.1% CHAPS), thoroughly resuspended using a vortex mixer, and then incubated at room temperature for 30 min. After centrifugation at 15,000 rpm at 4 °C for 30 min, the supernatant was collected. Protein concentration was determined using the Bradford assay. Each protein sample (200 μg) was digested with 50 μL trypsin solution at 37 °C overnight. iTRAQ proteomics was performed using a 4-plex procedure. The iTRAQ reagents 117, 118, 119 and 121 were used to label the peptides from 0 h, 12 h, 24 h and 48 h respectively (AB Sciex, Foster City, USA).

### 2DLC-MS/MS analysis

To reduce sample complexity for LC-MS/MS analysis, we separated the mixed peptides using nano-HPLC with the RP analytical column (Durashell-C18, 4.6 mm × 250 mm, 5 μm, 100 Å). Peptides were subsequently eluted using mobile phase A (98% ddH_2_O, 2% acetonitrile, pH 10) and mobile phase B (98% acetonitrile, 2% ddH_2_O, pH 10), the flow rate was maintained at 0.7 mL/min. The mixed peptides (desalted with 2% methyl alcohol and 0.1% formic acid) were further separated by nano-HPLC with the secondary RP analytical column (EASY-Spray column, 12 cm × 75 μm, C18, 3 μm). The RP mobile phase A was 100% H_2_O (with 0.1% formic acid); the RP mobile phase B was 100% acetonitrile (with 0.1% formic acid). The flow rate was maintained at 350 nL/min. Electrospray voltage of 2.1 kV versus the inlet of the mass spectrometer was used. MS was performed using a nano-LC coupled online to a QStar Elite mass spectrometer (Applied Biosystems, Foster City, USA), and raw data was collected using Analyst QS 2.0 controlling software (AB Sciex) through a nanospray ion source. ProteinPilot version 3.0 (AB Sciex) was used for processing the raw acquired iTRAQ data files.

### Data analysis

ProteinPilot version 3.0 (AB Sciex) was used for processing the raw acquired iTRAQ data files. Proteins were identified using the raw MS data in a search against the Swissprot-Uniprot protein database, with search parameters as follows: iTRAQ labeling at N-terminus and lysine residues, cysteine modification by carboxyamidomethylation and digestion by trypsin. For iTRAQ quantitation, the peptide for quantification was automatically selected by Pro Group^TM^ algorithm in ProteinPilot to calculate the reporter peak area, error factor (EF) and *p*-value. The false discovery rate (FDR) for each protein was estimated using a concatenated target-decoy database. The proteins were filtered with a FDR <0.01. The proteins were considered to be differentially expressed if their iTRAQ ratios were >1.5 or <0.666 in the viruliferous aphids compared with the nonviruliferous aphids (*p* <0.05).

### Functional analysis

The protein abundance data from Protein Pilot were further processed using the hierarchical cluster function of Gene Cluster software (http://bonsai.hgc.jp/~mdehoon/software/cluster/software.htm)^61^ under the default parameters with log-transformation. The functional annotation of identified differential proteins was performed using the information in the Uniprot database (http://www.uniprot.org)^62^ and DAVID bioinformatic resource (http://david.abcc. ncifcrf.gov/)^63^. The protein pathway annotations were analyzed using the KEGG database (http://www.kegg.jp/kegg/pathway.html)^64^.

### Verification of differentially expressed genes by RT-qPCR

Total RNAs were extracted using TRIzol reagent (Invitrogen, California, USA) from four groups of *R. padi*: collected after 0 h, 12 h, 24 h, or 48 h acquisition access period [AAP] on BYDV-GPV-infected oat plants). First-strand cDNA of each sample was synthesized using the FastQuant RT Kit (Tiangen, Beijing, China) and the kit protocol. Actin was used as the internal reference gene. The obtained cDNAs were used as the template for the q-PCR. The q-PCR reaction mixture comprised 6.4 μL RNase-free ddH_2_O, 10 μL 2× SuperReal PreMix Plus (SuperReal PreMix Plus kit [SYBR Green I, Tiangen]), 0.6 μL forward primer (10 mM), 0.6 μL reverse primer (10 mM), 0.4 μL 50× ROX Reference Dye, 2 μL diluted cDNA (1:30 dilution of cDNA sample template) or ddH_2_O (as the no-template control). The reaction program were as follows: 95 °C, 15 min, 1 cycle; followed by 40 cycles of 95 °C 10 s, 59.2 °C 32 s, 60 °C 32 s. Relative gene expression was calculated according to the Livak method (2^-ΔΔCt^). The experiments were repeated 3 times independently. Primers used in RT-qPCR for validation of differentially expressed genes are shown in Supplementary Table S1.

### Yeast two-hybrid (YTH) assay

#### Construction of BYDV-GPV RTP and CP bait fusion vectors

Total RNA was extracted from virus-infected wheat leaves using Trizol reagent. The genes encoding BYDV-GPV RTP and CP were RT-PCR-amplified respectively with the primer pair CP-pDHB1F/CP-pDHB1R and RTP-pDHB1F/RTD-pDHB1R (Supplementary Table S1), then the amplified products were purified and ligated with pMD19T simple vector (Takara, Dalian, China), and finally used to transform DH5a *E. coli* cells (Tiangen) according to the instructions. The positive clone was then confirmed by PCR analysis, sequencing and restriction enzyme digestion. The digested sequence was ligated into the SfiI-predigested pDHB1plasmid vector. To test auto-activation of the bait proteins, four sets of plasmid pairs, pDHB1-RTP/pOst1-NubI(pDHB1-CP/pOst1-NubI), pDHB1-RTDP/pPR3-N(pDHB1-CP/pPR3-N), pDHB1-largeT/pDSL-p53, pDHB1-largeT/pPR3-N were used to transform yeast strain NMY51 according to the manufacturer’s protocol (Dualsystems Biotech, Switzerland).

### Construction of *R. padi* cDNA library

The cDNA library of *R. padi* was constructed in prey plasmid pPR3-N using an EasyClone cDNA library construction kit (Dualsystems Biotech). Total RNA was extracted from *R. padi* using the Trizol reagent, and first- and double-strand cDNAs were synthesized according to the protocol of the Easy Clone cDNA library construction kit (Dualsystems Biotech). The amplified double-strand cDNA was digested using SfiI. To construct a cDNA library containing as many of the target genes as possible, we collected 200-1000-bp fragments. Then the cDNA fragments were ligated into the complementary sites of pPR3-N plasmid vector.

### Library screening

We used a DUAL hunter starter kit (Dualsystems Biotech) to screen *R. padi* cDNA library. Yeast strain NMY51 was sequentially transformed with the bait fusion vector (pDHB1-RTP or pDHB1-CP) and *R. padi* cDNA library using the lithium acetate method with single-stranded carrier DNA (ssDNA) according to the manufacturer’s protocol. The mixture was then spread onto TDO plates and incubated at 30 °C for 4 days. All potential positive hits were restreaked onto higher stringency QDO plates. The prey plasmids in positive clones were isolated using TIANprep yeast plasmid DNA kit (Tiangen).

### Positive prey analysis

The positive prey plasmids were further characterized by determining the DNA sequences of the inserts by sequencing. The sequencing results for the positive hits were used to search for reference sequences using BLASTX against the nonredundant (nr) NCBI protein database provided by NCBI (http://blast.ncbi.nlm.nih.gov/Blast.cgi) with default parameters. The functional and pathway annotation were done using the Uniprot database, DAVID bioinformatic resource and KEGG database.

### Retransformation of prey plasmid for interactors

To distinguish positive from false-positive interactions and further confirm the interaction of bait and prey proteins, we selected 22 positive library plasmids for RTP and 5 for CP based on their molecular function to cotransform yeast strain NMY51 with the prey plasmid and bait plasmid. At the same time, three sets of plasmids, pDHB1-largeT/pDSL-p53, pDHB1-RTD/pOst1-NubI (pDHB1-CP/pOst1-NubI) were respectively used to cotransform yeast strain NMY51 as positive controls, and pDHB1-largeT/pPR3-N were used to cotransformed yeast strain NMY51 as a negative control.

### Chemiluminescence Co-IP assay

For constructing the pAcGFP1-bait and pProLabel prey fusion protein expression vectors, the full-length coding sequence of RTP or CP was cloned and fused to pAcGFP1 vector to express the bait fusion protein. Each of the seven potential positive prey genes (pancreatic lipase-related protein, tropomyosin, twinstar, complexin, nascent polypeptide-associated complex subunit alpha, cytochrome c oxidase polypeptide IV-like and cytochrome P450 4g15) derived from the yeast two-hybrid assay was cloned into the pProLabel vector to be expressed as the prey fusion protein. The pAcGFP1-bait plasmid and pProLabel-prey plasmid were used to cotransfect mammalian cells (Human Embryonic Kidney 293 [HEK293] cells) using a CalPhos Mammalian Transfection Kit (Clontech, USA) according to the manufacturer’s protocol. All primers for the Co-IP assay are listed in Supplementary Table S1. Plasmid set pAcGFP1-p53/pProLabel-T was used for the positive control, and set pAcGFP1-Lam/pProLabel-T was used for the negative control. After 48 h, cells were lysed and incubated with anti-GFP polyclonal antibody (Clontech) for 2 h at 4 °C. Protein A/G agarose beads were added to each lysate, and the mixture was incubated with gentle rotation at 4 °C overnight. Beads were collected and washed nine times by centrifugation. Each sample of beads was then resuspended in the lysis/complementation buffer and then transferred to a well in a 96-well solid plate (Costar, USA) to determine the protein-protein interaction strength. To each sample, substrate mix was added, and pProLabel activity was measured using a GloMaxTM 96 Microplate Luminometer (Promega, Wisconsin, USA) after 0, 10, 15, 20, 25, 30 and 45 min.

### Construction of protein-protein interaction network

The protein-protein interaction network for all proteins identified by iTRAQ and YTH was created using String (http://string-db.org)^65^. Orphan proteins that were unconnected with other proteins were removed, and the network information was exported for visualization in Cytoscape v.3.1.1 (www.cytoscape.org)^66^.

## Additional Information

**How to cite this article**: Wang, H. *et al*. Integrative proteomics to understand the transmission mechanism of *Barley yellow dwarf virus-GPV* by its insect vector *Rhopalosiphum padi*. *Sci. Rep*. **5**, 10971; doi: 10.1038/srep10971 (2015).

## Supplementary Material

Supplementary Information

## Figures and Tables

**Table 1:** Differential proteins between healthy and viruliferous *Rhopalosiphum padi* as identified by iTRAQ.

			12 h AAP^c^ : 0 h AAP		24 h AAP : 0 h AAP		48 h AAP : 0 h AAP		
Protein and pathway	%Cov (95)^a^	Peptides (95%)^b^	Ratio^d^	Pval^e^	Ratio	PVal	Ratio	PVal	Accession
**Metabolic pathways**									
PREDICTED: glycerol-3-phosphate dehydrogenase, mitochondrial-like isoform 4	14.58	16	4.09	0.0204	0.92	0.7964	0.43	0.2628	gi|328709677
PREDICTED: glutamate dehydrogenase, mitochondrial-like	36.31	30	3.91	0.0004	2.11	0.0162	2.25	0.0503	gi|193582510
ATP synthase subunit beta, mitochondrial	55.47	75	3.22	0.0204	3.56	0.0155	0.76	0.5102	gi|209915626
putative mitochondrial ATP synthase alpha subunit precursor	49.00	63	3.56	0.0136	3.22	0.0204	0.96	0.3627	gi|52630965
PREDICTED: probable aconitate hydratase, mitochondrial-like	21.17	23	2.99	0.0195	1.11	0.3415	0.29	0.4397	gi|328716624
PREDICTED: acetyl-CoA carboxylase-like isoform 4	24.69	74	2.03	0.0075	2.11	0.0035	1.53	0.0795	gi|328714419
PREDICTED: glyceraldehyde-3-phosphate dehydrogenase-like	42.17	32	0.25	0.0353	1.13	0.3332	0.17	0.0047	gi|193688110
PREDICTED: glucosamine-fructose-6-phosphate aminotransferase [isomerizing] 2-like isoform 1	8.09	7	0.21	0.0464	0.58	0.1816	0.36	0.0977	gi|328718712
PREDICTED: elongation factor 2-like	31.64	49	0.16	0.0001	0.94	0.3257	0.33	0.0180	gi|193690671
ATP citrate lyase	41.21	60	1.28	0.7255	3.16	0.0238	2.19	0.1524	gi|237874159
peroxiredoxin-6-like	49.09	18	2.25	0.0613	2.29	0.0436	2.01	0.0231	gi|240848687
PREDICTED: NADH dehydrogenase [ubiquinone] flavoprotein 1, mitochondrial-like	13.28	7	0.94	0.8571	0.44	0.1259	0.06	0.0067	gi|193587168
**Ribosome**									
PREDICTED: 40 S ribosomal protein S8-like	46.15	21	0.23	0.0114	0.92	0.9471	0.69	0.5592	gi|328709805
40 S ribosomal protein S2-like	40.38	21	0.08	0.0397	1.24	0.8129	1.45	0.9157	gi|240849555
ribosomal protein S13e-like	29.14	5	0.17	0.0229	0.70	0.3303	1.29	0.7132	gi|242247489
uncharacterized protein LOC100165190	2.79	1	0.90	0.8412	1.04	0.9312	0.01	0.0453	gi|350535082
ACYPI010200	31.98	17	0.21	0.0281	0.67	0.7261	0.44	0.4620	gi|239789423
**Regulation of actin cytoskeleton**									
PREDICTED: vinculin-like	18.15	17	1.42	0.0361	1.67	0.0350	1.61	0.0497	gi|193606151
PREDICTED: myosin heavy chain, muscle isoform 1	49.72	233	9.64	4.43E-05	5.40	1.06E-05	1.75	0.1814	gi|328702403
**Signaling pathway**									
PREDICTED: voltage-dependent anion-selective channel-like isoform 1	25.27	10	0.95	0.3256	1.32	0.3841	0.17	0.0160	gi|193690508
putative activated protein kinase C receptor	54.86	24	0.54	0.0629	0.58	0.0984	0.45	0.0496	gi|52630921
**Protein processing**									
PREDICTED: heat shock protein 83-like	44.37	63	0.31	0.0867	0.58	0.0554	0.28	0.0450	gi|193652748
PREDICTED: translocon-associated protein subunit gamma-like	5.95	2	1.07	0.8883	1.14	0.7876	0.01	0.0498	gi|328698093
**Synaptic vesicle cycle**									
vesicle-associated membrane protein-like	31.43	4	0.01	0.0478	0.86	0.7880	0.38	0.2442	gi|240848629
**RNA degradation**									
symbionin symL-pea aphid	39.05	56	0.28	0.0021	0.47	0.1640	0.13	0.0173	gi|285430
**Unknown**									
myofilin isoform a	42.59	16	5.30	0.0365	2.78	0.2643	2.56	0.1109	gi|253735723
PREDICTED: paramyosin, long form-like	44.71	81	2.54	0.0027	1.89	0.0377	0.63	0.9907	gi|328724595
PREDICTED: staphylococcal nuclease domain-containing protein 1-like	34.02	49	0.43	0.0047	0.65	0.2712	0.43	0.0128	gi|193688302
cuticular protein 62 precursor	32.87	9	2.13	0.1359	0.44	0.0262	0.60	0.1887	gi|288558725
PREDICTED: KH domain-containing, RNA-binding, signal transduction-associated protein 3-like	20.23	10	0.77	0.0560	0.85	0.6793	0.63	0.0059	gi|193688146
putative histone h4-like protein, partial	52.48	23	1.20	0.4071	0.96	0.8159	0.62	0.0376	gi|604788259
glutathione S-transferase delta 1	26.56	6	0.51	0.1068	1.64	0.3014	0.25	0.0351	gi|392584108
PREDICTED: hypothetical protein LOC100164538	46.15	67	0.24	0.1818	0.74	0.6521	0.02	0.0481	gi|193706873

^a^Percentage of matching amino acids from identified peptides having confidence ≥95%.

^b^Number of distinct wpeptides having at least 95% confidence.

^c^Acquisition access period (AAP) on BYDV-GPV-infected wheat plants.

^d^Fold-change between two samples, ratio >1 represented up-regulation, ratio <1 represented down-regulation.

^f^Evaluation of significance of fold-change.

**Table 2:** Putative proteins of *Rhopalosiphum padi* that interacted with the CP or RTP of *Barley yellow dwarf virus-GPV* in a yeast two-hybrid assay.

Protein description and pathway	e-Value	Identity	Accession
**Metabolic pathways**			
V-type proton ATPase subunit d^b^	2.00E-34	98%	NP_001191854.1
Pancreatic lipase-related protein^ab^	9.70E-02	32%	EGI64115.1
Ubiquitin carboxyl-terminal hydrolase^b^	1.00E-105	81%	XP_008186680.1
**Ribosome**			
ribosomal protein L30-like protein^b^	3.00E-07	100%	NP_001119630.1
60 S ribosomal protein L8-like^b^	2.4	100%	NP_001155749.1
**Regulation of actin cytoskeleton**			
Twinstar^b^	1.00E-46	99%	NP_001119642.1
**Signaling pathway**			
guanine nucleotide-binding protein G(s) subunit alpha^b^	0.032	100%	XP_001944148.1
**Protein processing**			
t-complex protein 1 subunit theta-like^b^	0.32	100%	XP_001944638.1
**Synaptic vesicle cycle**			
complexin^b^	1.00E-25	99%	NP_001156645.1
**Neuroactive ligand-receptor interaction**			
neuronal acetylcholine receptor subunit alpha-7-like^b^	2.00E-35	97%	XP_001945224.2
glutamate receptor ionotropic kainate 3-like isoform X3^b^	3.00E-56	98%	XP_003242706.1
**Proteasome**			
proteasome beta 3 subunit-like^b^	3.00E-147	98%	NP_001155482.1
**Cardiac muscle contraction**			
tropomyosin^b^	6.00E-91	85%	XP_008178569.1
**Ribosome biogenesis in eukaryotes**			
H/ACA ribonucleoprotein^a^	1.00E-24	96%	XP_003248969.1
**Unknown**			
cytochrome P450 4g15^b^	4.00E-31	94%	XP_001944205.2
cytochrome c oxidase polypeptide IV-like^ab^	5.00E-109	93%	NP_001156047.1
nascent polypeptide-associated complex subunit alpha^ab^	1.00E-94	99%	NP_001156301.1
cytochrome c oxidase subunit Va-like^b^	3.00E-97	89%	NP_001156232.1
cuticular protein 5 precursor^b^	6.00E-40	97%	NP_001156154.1
chemosensory protein-like precursor^b^	1.00E-58	94%	NP_001119652.1
coiled-coil-helix-coiled-coil-helix domain containing^ab^	3.00E-35	97%	NP_001155450.1
titin-like^b^	0	86%	XP_008188078.1
fibronectin type 3 domain^b^	3.00E-05	69%	XP_008180552.1
serine/arginine repetitive matrix protein 1^ab^	2.00E-10	97%	XP_003242057.1
Cu(I)-responsive transcriptional regulator^b^	3.00E-14	75%	XP_001948052.2
jagunal homolog^b^	4.00E-44	97%	NP_001155727.1
mitochondrial import inner membrane translocase subunit Tim22 isoform X1^a^	9.00E-71	95%	XP_003245191.1
EMI domain-containing protein^a^	2.00E-60	85%	XP_001948474.2

^a^Prey proteins identified by bait BYDV-GPV CP.

^b^Proteins identified by bait BYDV-GPV RTP.

^ab^Prey proteins identified by both bait BYDV-GPV CP and RTP.
