# Supplementary material for: Integrative proteomics to understand the transmission mechanism of Barley yellow dwarf virus-GPV by its insect vector Rhopalosiphum padi
Source: Sci Rep. 2015 Jul 10;5:10971. doi: 10.1038/srep10971 (PMC4498328; doi:10.1038/srep10971)
Supplement: Supplementary Information [file srep10971-s1.pdf]

Supporting Information for

Integrative proteomics to understand the transmission mechanism of *Barley yellow dwarf virus*-GPV by its insect vector *Rhopalosiphum padi*

Hui Wang[1], Keke Wu[1,2], Yan Liu[1], Yunfeng Wu[2], Xifeng Wang[1*]

[1]*State Key Laboratory for Biology of Plant Diseases and Insect Pests, Institute of Plant Protection, Chinese Academy of Agricultural Sciences, Beijing, China*

[2]*College of Plant Protection, Northwest A & F University, Yangling 712100, China*

*Corresponding author: Email: xfwang@ippcaas.cn

Supplementary Table S1 | Primers used in this study

Primer	Primer sequence
**RT-qPCR**	
Myosin-F	GGTCGTAATAAGGACCAAGTTGC
Myosin-R	CCTCACGTTGGTACTCTTCTTG
Paramyosin-F	CAGATGAACGTTACCAACGG
Paramyosin-R	GCGTTGGCTTCGACTTCTTC
Vinculin-F	GCTCTAAATCAAGCTGCCACAC
Vinculin-R	CATCTGGTCCTCAGATAATCG
Actin-F	AACGGAAGCACCTTTGAACC
Actin-R	GGAAGAAGCAGCAGTAGCCAT
**Yeast two-hybrid assay**	
CP-pDHB1F	ATTAACAAGGCCATTACGGCCATGAGTACGGTCGCCCTTAG
CP-pDHB1R	AACTGATTGGCCGAGGCGGCCCCCTATTTCGGGTTTTGAAACAGG
RTP-pDHB1F	ATTAACAAGGCCATTACGGCCGTAGACGCGGAACCCGGTCCTAG
RTP-pDHB1R	AACTGATTGGCCGAGGCGGCCCCTTAGGAACGGCCACCACCAAATG
**Co-IP assay**	
CP-pAcGFPF	TCTGTCGACGAGGAGCAGAAGCTGATCTCAGAGG
CP-pAcGFPR	CGCAAGCTTTTATTTCGGGTTTTGAAACAGGAC
RTP-pAcGFPF	TCTGTCGACGAGGAGCAGAAGCTGATCTCAGAGG
RTP-pAcGFPR	CGCAAGCTTTTAGGAACGGCCACCACCAAATG
Pan-pProLabelF	TCTGTCGACCCATACCCATACGATGTTCCAGAT
Pan-pProLabelR	TTATCTAGATTATCTAATGTCTTCTTTGGATA
Tsr-pProLabelF	GCAGTCGACATGGCATCGGGAGTAACCGTAG
Tsr-pProLabelR	GGTGGATCC CTGTCTGTCGGTGGACCTGAG
P450-pProLabelF	GACGGTACCACACTCTTCAATGTCAACAGCCGTG
P450-pProLabelR	GGTGGATCCAGCAGCGGTCTTAGCTAGTGAC
Tpm-pProLabelF	GACGGTACCAAGAAGGGTTTCATGACCCCGGAC
Tpm-pProLabelR	TTA TCTAGA AGCTGTTGCTGCTGGTTCCGGTACG
CV4-pProLabelF	GCAGTCGACGTACAGGGTGTGATGCAAGTGC
CV4-pProLabelR	GGTGGATCCACTTTTCCATGTGTTCTTTTCGTAATC
Naca-pProLabelF	GCAGTCGACATGCCTGAATTAACGGAAATC
Naca-pProLabelR	GGTGGATCCCATTGTCAAGTCCATAATAGC
Comx-pProLabelF	GCAGTCGACATGGCGGCTTTTGTGGCAAAGC
Comx-pProLabelR	GGTGGATCCTTGAAGACTGCACTTGCTCTC

Supplementary Figure S1 | Expression verification of the bait fusion vector. Expression of BYDV-GPV RTP bait and the CP bait were tested using the DUAL hunter functional assay. BYDV-GPV CP or RTD bait fusion vector was used to cotransformed with the control plasmid pOst1-NubI or pPR3-N empty insert and grown on selective SD medium (DDO/TDO/QDO). Co-expression of the pDHB1-CP (A)/RTP (B) with Ost1-NubI resulted in reporter gene activation as shown by growth of the yeast transformants on QDO, while co-expression of pDHB1-CP (A)/RTD (B) with pPR3-N empty insert did not yield any yeast transformant growth on QDO. pDHB1-LargeT and pDSL-p53 were used as positive controls; pDHB1-LargeT and pPR3-N-empty insert were used as negative controls.

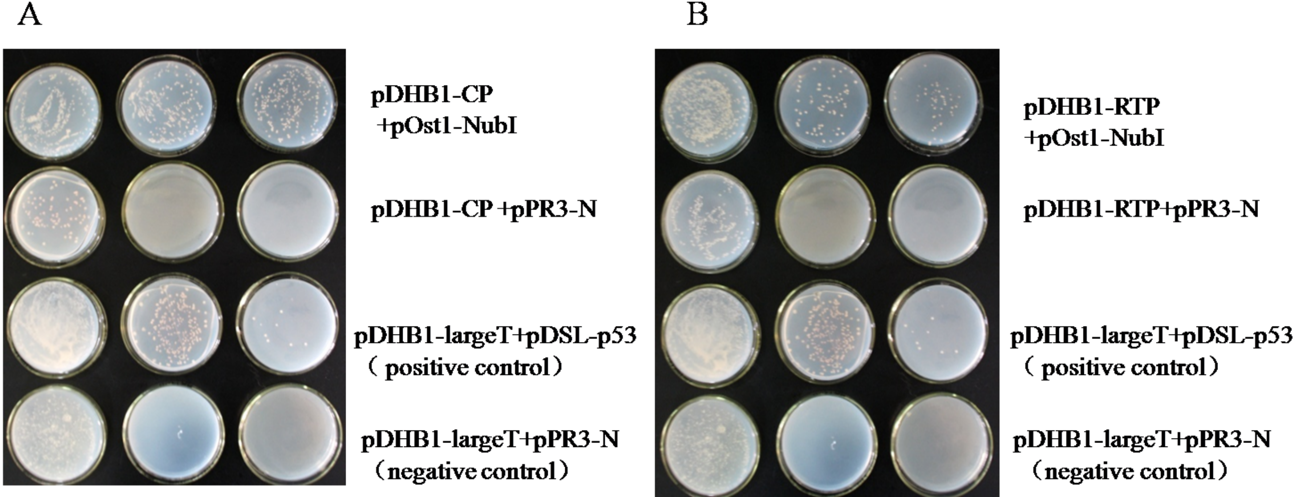
A
pDHB1-CP
+pOst1-NubI
pDHB1-CP +pPR3-N
pDHB1-largeT+pDSL-p53
（positive control）
pDHB1-largeT+pPR3-N
（negative control）
B
pDHB1-RTP
+pOst1-NubI
pDHB1-RTP+pPR3-N
pDHB1-largeT+pDSL-p53
（positive control）
pDHB1-largeT+pPR3-N
（negative control）
